# Acute Promyelocytic Leukemia Presenting as Spontaneous Abortion Amidst Tropical Fever Season: A Diagnostic Challenge

**DOI:** 10.7759/cureus.99395

**Published:** 2025-12-16

**Authors:** Ambadas Rathod, Monica Gupta, Anshu Palta

**Affiliations:** 1 General Medicine, Government Medical College and Hospital, Chandigarh, Chandigarh, IND; 2 Pathology, Government Medical College and Hospital, Chandigarh, Chandigarh, IND

**Keywords:** acute myeloid leukemia (aml), acute promyelocytic leukemia (apl), bleeding per vagina, pancytopenia, spontaneous abortion

## Abstract

Acute myeloid leukemia (AML) is a hematologic malignancy with diverse and often nonspecific clinical manifestations. This report discusses a 30-year-old female presenting with fever for six days and per vaginal (PV) bleeding persisting for three days during the tropical fever season. Initial evaluation indicated pancytopenia, leading to considerations of infectious, nutritional, or hematologic etiologies. A detailed history revealed 10 weeks of amenorrhea with a positive urine pregnancy test (UPT) confirmed pregnancy, which was further supported by ultrasonography (USG), suggesting incomplete abortion. However, over the next few hours, she passed multiple clots per vagina, and a repeat USG confirmed that she had sustained a complete abortion. Despite multiple transfusions, pancytopenia persisted, prompting a bone marrow examination that confirmed acute promyelocytic leukemia (APL), a subtype of AML. This case underscores that unexplained pancytopenia persisting beyond seven days after pregnancy loss warrants early bone marrow evaluation to rule out hematologic malignancies, as AML can present with gynecological symptoms in young females and requires prompt diagnosis.

## Introduction

Acute myeloid leukemia (AML) accounts for 10-15% of acute leukemias but often presents with nonspecific symptoms. In addition, data on cases manifesting primarily with gynecologic complaints in young women are limited, potentially leading to delayed diagnosis. AML during pregnancy is rare, occurring in roughly 1 in 75,000 to 1 in 100,000 pregnancies [1]. Because these presentations often mimic common acute undifferentiated fevers, especially in tropical regions, clinicians must maintain a high index of suspicion and pursue a thorough hematologic evaluation when standard workups fail to identify an infectious etiology. When early pregnancy-related bleeding occurs in the setting of febrile illness during the tropical fever season, hematologic malignancies may be overlooked, leading to potentially fatal diagnostic delays.

## Case presentation

A 30-year-old, previously healthy woman presented with fever for 6 days and bleeding per vagina (PV) for 3 days. There was no history of rash, joint pains, retro-orbital pain, or abdominal pain. There was no history of bleeding from any other site, except bleeding PV, or a past history of similar complaints. On examination, she was febrile. Blood pressure was 95/60 mmHg, heart rate was 104 beats/ minute, with saturation of 98% under room air. Pallor was present on examination of the lower palpebral conjunctiva, but the remaining examination was normal. There was no gum hypertrophy, rash, or petechiae.

Her baseline investigations revealed pancytopenia, with hemoglobin (Hb) of 6.6 g/dL, total leucocyte count (TLC) of 3.3 × 10⁹/L, and platelets of 8 × 10⁹/L. The initial peripheral blood film showed microcytes, macrocytes, anisopoikilocytosis, and markedly reduced platelets. Because her symptoms began during the tropical disease season in India (October-November), an initial evaluation for dengue, malaria, scrub typhus, and leptospirosis was performed, all of which were negative. Both blood and urine cultures were sterile. Renal and liver function tests, as well as coagulation parameters, were normal. Disseminated intravascular coagulation (DIC) workup, including fibrin degradation products (FDP), D-dimer, and fibrinogen, was unremarkable. Nutritional deficiencies were excluded by normal iron studies, vitamin B12, and folate levels (Table 1).

**Table 1:** Lab Investigations during hospital stay Ig- Immunoglobulin, NS1- Non-Structural Protein 1, HBsAg- Hepatitis B surface antigen, HCV- Hepatitis C Virus, HIV- Human Immunodeficiency Virus, SGOT- Serum Glutamic Oxaloacetic Transaminase, SGPT- Serum Glutamic Pyruvic Transaminase, TIBC- Total Iron Binding Capacity

Parameter	Day 1	Day 2	Day 3	Day 5	Day 7	Day 9	Normal range
Hemoglobin	6.6	7.6	6.6	7.6	10.1	9.1	12-16 g/dL
Total leucocyte count	3.3	3.2	3.72	3.89	3.86	2.3	4-7 x 10^9^ /L
Platelets	8	10	12	5	13	11	150-450 x10^9^ /L
Mean corpuscular volume	96.2	97.4	95	98	96.5	97.1	83-101 fL
Corrected reticulocyte count	1	1.2		0.8		1.1	0.5-2.5%
Vitamin B12	598	-	-	-	-	-	211-900 ng/mL
Folic acid	5	-	-	-	-	-	3.5-5.5ng/mL
Iron	78	-	-	-	-	-	60-180 ug/ dL
Ferritin	689.5	-	-	-	-	-	22-322 ng/ mL
TIBC	258	-	-	-	-	-	240-450 ug/ dL
Transferrin saturation	30.2 %	-	-	-	-	-	15-45%
FDP	3.5	-	-	-	-	-	<10 ug/mL
D-dimer	464	-	-	-	-	-	<500 ng/mL
Fibrinogen	190 mg/dl	-	-	-	-	-	170-330 mg/dL
Prothrombin time	15	-	-	-	-	-	12-15 seconds
Activated partial thromboplastin time	34	-	-	-	-	-	28-32 seconds
Prothrombin time index	93%	-	-	-	-	-	80-100%
International normalized ratio	1.08	-	-	-	-	-	0.8-1.1
Dengue NS1 and dengue IgM	Negative Negative	-	-	-	-	-	Negative
Scrub typhus IgM	Negative	-	-	-	-	-	Negative
Rapid Malarial Antigen test	Negative	-	-	-	-	-	Negative
Leptospira IgM	Negative	-	-	-	-	-	Negative
HBsAg	Negative	-	-	-	-	-	Negative
HCV	Negative	-	-	-	-	-	Negative
HIV	Negative	-	-	-	-	-	Negative
Urea	17	-	-	-	-	-	15-45 mg/dL
Creatinine	0.8	-	-	-	-	-	0.8-1.2 mg/dL
Bilirubin	0.7	-	-	-	-	-	0.2-1.0 mg/dL
SGOT	37	-	-	-	-	-	<40 IU/L
SGPT	21	-	-	-	-	-	<40 IU/L

**Figure 1:**
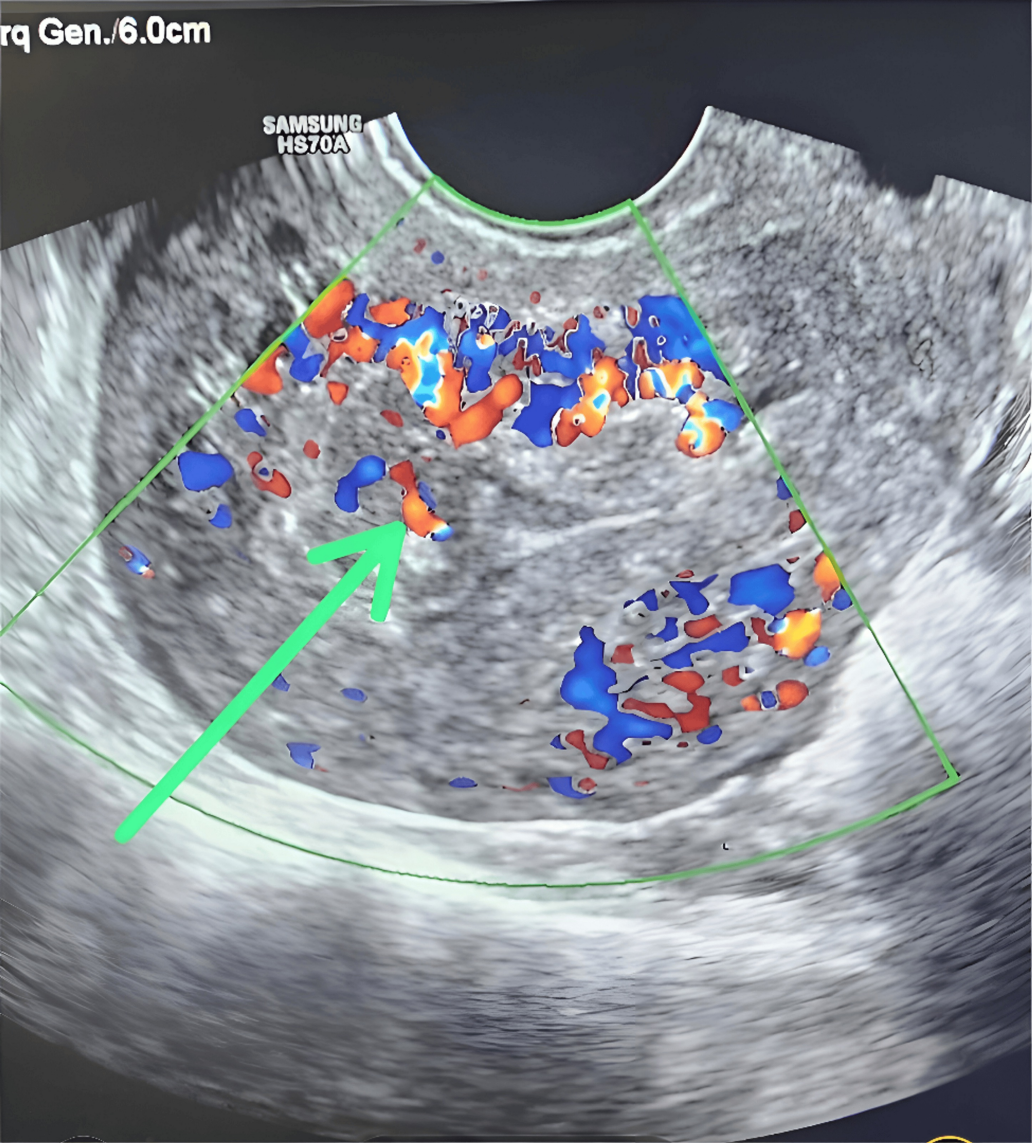
Transvaginal color Doppler ultrasound demonstrating marked irregular and thickened heterogeneous material within the uterine cavity, as shown by the green arrow, with prominent internal vascularity

Further history revealed 10 weeks of amenorrhea and a positive urine pregnancy test. In view of this, an incomplete abortion was suspected, and a gynecological consultation was obtained. Transvaginal ultrasonography revealed retained products of conception (Figure 1), consistent with an incomplete abortion, while abdominal USG showed no evidence of organomegaly.

Within a few hours, the patient passed clots per vaginum. A repeat gynecological review and follow-up ultrasound demonstrated an empty uterus, confirming spontaneous evacuation of the products of conception.

**Figure 2:**
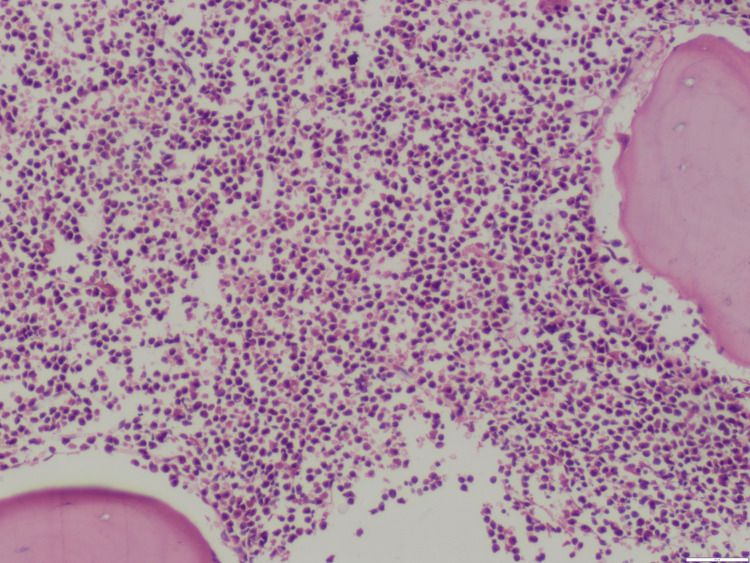
Hematoxylin and eosin (H&E)-stained bone marrow biopsy showing increased hypercellularity (magnification 10x)

**Figure 3:**
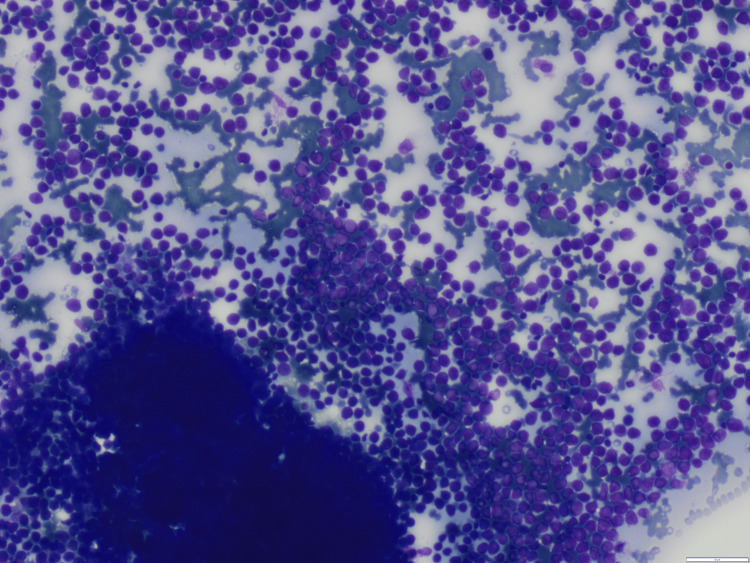
Giemsa stain of the bone marrow aspirate demonstrating numerous blasts with prominent nucleoli and scant cytoplasm (magnification 10x)

**Figure 4:**
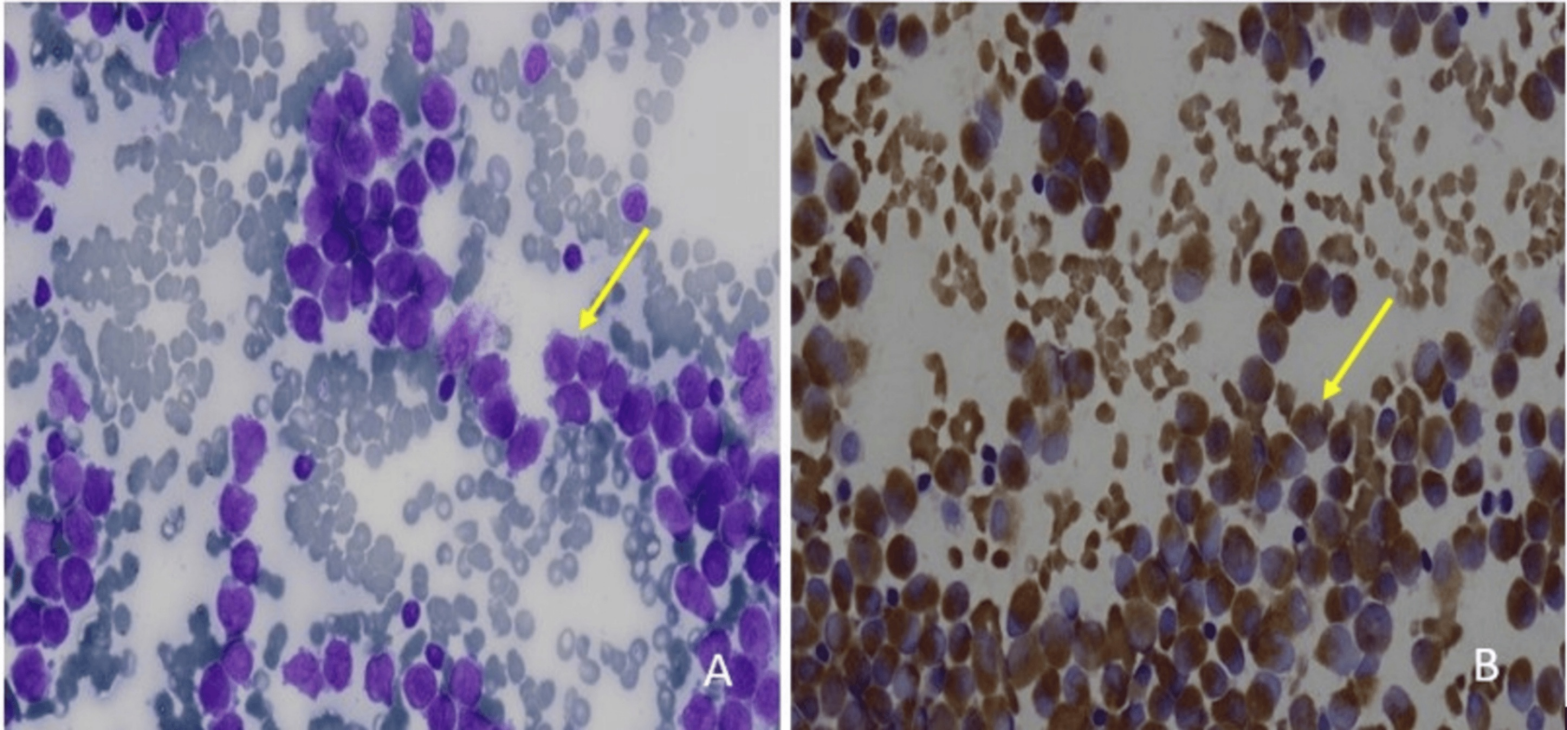
Panel A: Bone marrow aspirate smear showing numerous blasts with high nucleocytoplasmic ratio, fine chromatin, and prominent nucleoli as highlighted by the yellow arrow (Magnification 100x). Panel B: Myeloperoxidase (MPO) cytochemical stain demonstrating strong granular cytoplasmic positivity in the majority of blasts as highlighted by the yellow arrow (Magnification 100x).

The patient was managed conservatively with intravenous (IV) fluids and empiric broad-spectrum antibiotics: doxycycline 100 mg IV twice daily, ceftriaxone one g IV two times daily, and metronidazole 500 mg IV three times daily, initiated to provide coverage for suspected post-abortion pelvic infection despite negative initial culture results. She was transfused four packed cells, 10 random, and two single donor platelets. Although her PV bleeding resolved and she became afebrile, pancytopenia persisted. Despite spontaneous abortion and adequate transfusion support, cytopenias continued for more than seven days, prompting further evaluation with bone marrow examination. The marrow was hypercellular with a predominance of myeloperoxidase (MPO)-positive promyelocytes, leading to a provisional diagnosis of acute leukemia, most consistent with APL (Figures 2-4). The patient was referred to a higher center hemato-oncology department due to the unavailability of the same at our center, where she was found to be positive for PML RARA by reverse transcription-polymerase chain reaction (RT-PCR), confirming the diagnosis of APL.

She was started on all-trans-retinoic acid (ATRA) 45 mg/m² and arsenic trioxide (ATO) 0.15 mg/kg. Post-induction bone marrow was hypercellular, without promyelocytes or blasts. Her all cell lines improved, and pancytopenia resolved; she was discharged home on ATRA 30 mg orally twice daily, acyclovir 400 mg peroral twice daily, fluconazole 400 mg once daily, co-trimoxazole 800 mg/160 mg once daily, along with multivitamins. This case demonstrates the diagnostic challenge due to the atypical and rare presentation of APL as early pregnancy loss.

## Discussion

The majority of cases of AML in pregnancy are diagnosed in the second (37%) and third trimesters (40%), with only ~23% detected in the first trimester, likely underestimated due to early pregnancy losses. The present case falls within this underreported subset of first-trimester APL diagnoses. A systematic review of 245 AML cases in pregnancy, including 92 APL cases, similarly emphasized the predominance of diagnoses in later trimesters, with fewer first-trimester detections likely influenced by underreporting [2-4]. AML should be suspected in pregnant patients with marked cytopenias or leukocyte abnormalities and >20% blasts on peripheral smear or bone marrow. Besides obstetrics, AML may very rarely present as myeloid sarcoma in the gynecologic tract or as uterine relapse of AML [5]. An unusual feature in this case was the absence of disseminated intravascular coagulation despite profound cytopenias, a hallmark complication of APL. This atypical presentation likely contributed to diagnostic delay and underscores the need for heightened suspicion even when classic features are absent.

APL is a rare, invasive AML subtype (prevalence 0.23/100,000). Cytogenetically, a balanced translocation t (15;17) (q24; q21) produces the PML-RARA fusion gene [6]. This hybrid gene gives this once-fatal leukemia a special sensitivity to differentiation agents like ATRA and ATO as well as anthracycline-based chemotherapy, making the disease highly curable (cure rates of about 90%) [7].

In pregnancy, APL is typically diagnosed at a median maternal age of 30 years, and the median period of gestation is 25 weeks. In a systematic review of 96 pregnant women with APL by Santolaria et al., the following cases were reported across all trimesters: 16 in the first, 46 in the second, 29 in the third, 4 postpartum, and 1 case had unclear gestational age [8]. APL diagnosis in pregnancy mirrors that in non-pregnant patients; bone marrow aspiration and biopsy are safe, with >30% promyelocytes confirming diagnosis, while molecular tests, peripheral smear, and flow cytometry provide supportive evidence. Hemorrhage is the major cause of early mortality in APL [9]. Maternal APL increases risks of preterm birth, intrauterine growth restriction, abortion, perinatal mortality, and complications such as placental abruption, inflammation, and infection [10].

Managing AML during pregnancy is challenging due to the need to balance maternal and fetal outcomes. Santolaria et al. have treated their patients with induction therapy that included ATRA alone in 32% or combined with chemotherapy in 44%, while 22% received anthracycline-based chemotherapy. Arsenic trioxide (ATO)-based regimens were administered in three recent cases (2016-2019) following stillbirth at 26 weeks or delivery of healthy infants at 35 and 39 weeks [8]. However, the European Leukemia Net (ELN) guidelines advise avoiding arsenic trioxide in pregnancy. In the second to third trimesters, ATRA alone or ATRA with chemotherapy may be used; if chemotherapy is deferred, it should start immediately after delivery [7].

APL is treated with ATRA plus anthracycline chemotherapy with high cure rates, but pregnancy complicates management due to teratogenic and cardiac risks. These drugs are contraindicated in the first trimester; treatment delay must be balanced against fetal harm. After the first trimester, ATRA and idarubicin can be used with caution, though risks of fetal growth restriction and cardiac issues, including reversible arrhythmias, persist, requiring close fetal monitoring [11]. In this case, spontaneous abortion, while an unfortunate outcome, resolved the ethical dilemma of balancing fetal teratogenic risk against maternal mortality, allowing the initiation of standard ATRA- and ATO-based therapy.

Maternal outcomes in APL during pregnancy were overall favorable, with high survival and remission rates across all trimesters. Survival improved with later gestational age at diagnosis, with 21 of 22 mothers surviving in the first trimester, 29 of 38 in the second, and 29 of 37 in the third, and remission rates exceeding 90% in all groups [11]. Fetal outcomes in 97 APL pregnancies varied markedly, with reduced fetal survival, especially earlier in gestation. Overall, fetal survival increased significantly as pregnancy advanced [11].

In the setting of adults attending a hematology clinic with persistent pancytopenia (>7-10 days), acute leukemia is documented in 17.9% cases [12]. However, hematological malignancy is expected in less than 1% of tropical fever cases. The threshold for bone marrow should be low when patients do not have a clear diagnosis after the initial non-invasive workup, have atypical symptoms or a lack of typical features for local endemic diseases, fail to respond clinically and hematologically to appropriate initial management, or have persistent pancytopenia (beyond 7-10 days) despite the presumptive treatment for common causes.

## Conclusions

This case underscores the necessity of a comprehensive hematologic workup in patients presenting with persistent pancytopenia once infectious causes are excluded. Clinicians should maintain a high suspicion for AML and its rare presentations in such settings. In our case, the patient’s initial evaluation focused on tropical infections, nutritional causes, sepsis, and DIC, all of which were ruled out. Persistent cytopenias beyond seven days despite supportive management prompted further investigation, with bone marrow examination ultimately establishing the diagnosis of APL. Early and timely bone marrow evaluation in cases of pancytopenia persisting beyond 7-10 days, along with heightened diagnostic vigilance, particularly during the tropical fever season, is crucial to prevent potentially life-threatening delays in diagnosing atypical presentations of leukemia.
